# Supplementary material for: Prioritization of regulatory variants with tissue-specific function in the non-coding regions of human genome
Source: Nucleic Acids Res. 2021 Oct 14;50(1):e6. doi: 10.1093/nar/gkab924 (PMC8754628; doi:10.1093/nar/gkab924)
Supplement: gkab924_Supplemental_File [file gkab924_supplemental_file.pdf]

Supplemental Materials

for

Prioritization of regulatory variants with tissue-specific function in the non-coding regions of human genome

Shengcheng Dong[1] and Alan P. Boyle[1,2*]

1. Department of Computational Medicine and Bioinformatics, University of Michigan, Ann Arbor, MI

2. Department of Human Genetics, University of Michigan, Ann Arbor, MI

Supplemental Tables

Supplemental Table S1. Number of allele specific TF binding (ASB) training SNVs in 6 cell lines.

Cell type name	# of ASB SNVs	# of control SNVs
GM12878	1,848	12,382
A549	215	1,357
H1hESC	1,464	6,525
HepG2	2,717	24,479
K562	767	12,635
MCF7	653	8,034

Supplemental Table S2. Feature list in random forest models of TURF.

**Generic features**	
TF binding sites from ChIP-seq	Binary variable
DNase I hypersensitive sites from DNase-seq	Binary variable
DNase footprints	Binary variable
DNase footprints with matched TF ChIP-seq peaks	Binary variable
TF motifs from PWM matching	Binary variable
TF motifs from PWM matching with matched TF ChIP-seq peaks	Binary variable
Information content change of two alleles in PWM matching	Numerical variable
Information content change of two alleles in PWM matching with matched TF ChIP-seq peaks	Numerical variable
eQTLs	Binary variable
Quantiles (25%,50%,75% and 100%) and variance of ChIP-seq signals across all available ChIP-seq experiments from ENCODE	Numerical variables
Functional significance score from *DeepSEA*	Numerical variable
**Tissue-specific features (in final ensemble model)**	
H3K4me1 peaks from ChIP-seq	Binary variable
H3K4me3 peaks from ChIP-seq	Binary variable
H3K27ac peaks from ChIP-seq	Binary variable
H3K36me3 peaks from ChIP-seq	Binary variable
H3K27me3 peaks from ChIP-seq	Binary variable
DNase I hypersensitive sites from DNase-seq	Binary variable
DNase footprints	Binary variable

Supplemental Table S3. Feature importance of TURF generic scores. The importance is calculated based on mean decrease of impurity from the random forest model.

**Feature name**	**Importance**
CHIP	0.124607
DNASE	0.033935
PWM	0.018686
FOOTPRINT	0.008609
EQTL_2	0.004167
PWM_matched	0.005055
FOOTPRINT_matched	0.001454
IC_change	0.050415
IC_matched_change	0.021396
funsig	0.122206
ChIP_var	0.149313
ChIP_max	0.167037
ChIP_quantile1	0.097849
ChIP_quantile2	0.090261
ChIP_quantile3	0.105011

Supplemental Table S4. Comparison of performance on cell type-specific predictions for MPRA variants and ASB SNVs.

Method	AUPR	AUROC	Correlation
**Test on MPRA variants in E116 (GM12878)**			
TURF_Tissue-Specific	0.548	0.731	0.450
TURF_Generic	0.536	0.740	0.447
GenoNet	0.536	0.723	0.442
DeepSEA	0.451	0.671	0.115
GenoSkyline	0.394	0.673	0.352
CADD	0.240	0.566	0.082
TURF_Ensemble (trained from ASB SNVs)	0.484	0.724	0.393
**Test on MPRA variants in E118 (HepG2)**			
TURF_Tissue-Specific	0.571	0.765	0.423
TURF_Generic	0.502	0.733	0.372
GenoNet	0.572	0.757	0.429
DeepSEA	0.401	0.667	0.166
GenoSkyline	0.326	0.628	0.225
CADD	0.332	0.607	0.152
TURF_Ensemble (trained from ASB SNVs)	0.479	0.728	0.357
**Test on MPRA variants in E123 (K562)**			
TURF_Tissue-Specific	0.441	0.681	0.344
TURF_Generic	0.400	0.664	0.294
GenoNet	0.420	0.706	0.340
DeepSEA	0.290	0.607	0.099

GenoSkyline	0.284	0.659	0.274
CADD	0.212	0.539	0.046
TURF_Ensemble (trained from ASB SNVs)	0.329	0.676	0.253
**Test on ASB SNVs in GM12878**			
TURF_Ensemble	0.511	0.894	0.538
TURF_Tissue-Specific	0.510	0.882	0.537
TURF_Generic	0.284	0.822	0.390
GenoSkyline	0.246	0.743	0.340
DeepSEA	0.222	0.688	0.190
GenoNet	0.129	0.685	0.158
**Test on ASB SNVs in HepG2**			
TURF_Ensemble	0.355	0.857	0.406
TURF_Tissue-Specific	0.353	0.831	0.401
TURF_Generic	0.221	0.797	0.323
DeepSEA	0.226	0.611	0.138
GenoSkyline	0.212	0.582	0.133
GenoNet	0.161	0.654	0.134
**Test on ASB SNVs in K562**			
TURF_Ensemble	0.316	0.847	0.376
TURF_Tissue-Specific	0.313	0.815	0.366
TURF_Generic	0.193	0.782	0.301
GenoNet	0.140	0.644	0.113
GenoSkyline	0.090	0.628	0.144
DeepSEA	0.062	0.572	0.054

Test on ASB SNVs in A549

TURF_Ensemble	0.313	0.872	0.369
TURF_Tissue-Specific	0.315	0.833	0.369
TURF_Generic	0.158	0.796	0.256
DeepSEA	0.052	0.760	0.119
GenoNet	0.030	0.690	0.094
GenoSkyline	0.022	0.632	0.049

Supplemental Figures

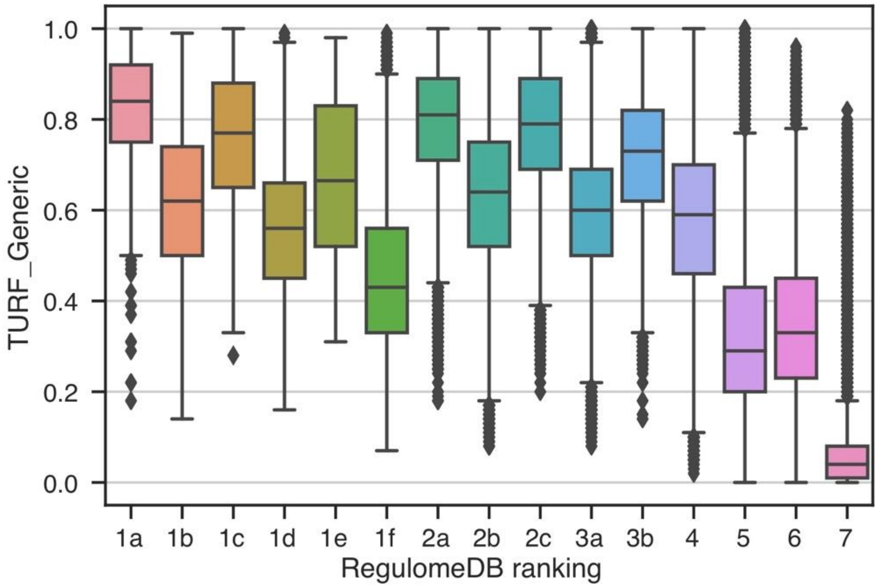

Supplemental Figure S1. Boxplot of TURF generic scores VS RegulomeDB ranking scores on 10,422,004 common SNVs from dbSNP153. X axis represents the original ranking scores from RegulomeDB v1.1, y axis represents the TURF generic scores from random forest model.

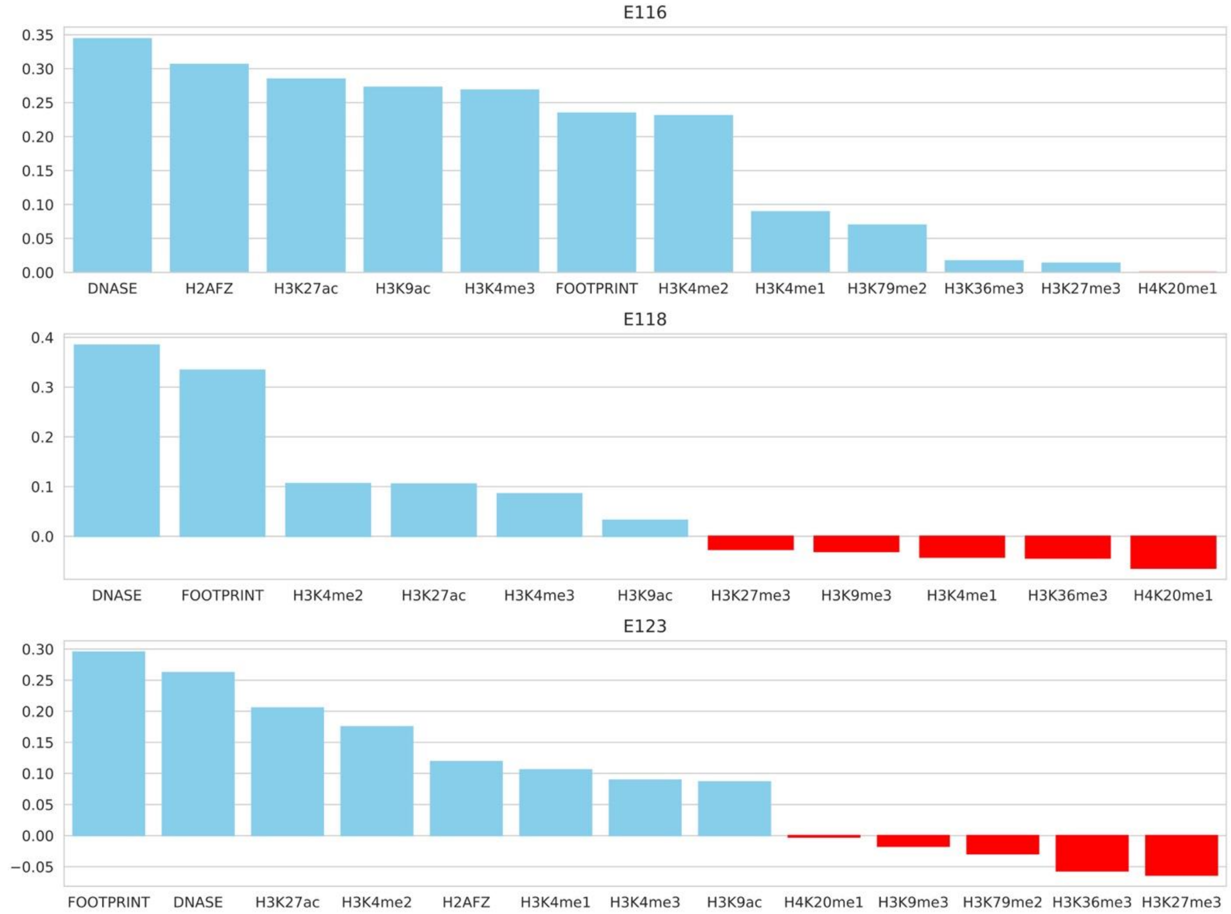

Supplemental Figure S2. Pearson correlation of labels and tissue-specific features in three MPRA datasets (E116: GM12878; E118: HepG2; E123: K562). Blue bars represent positive correlations while red bars represent negative correlations.

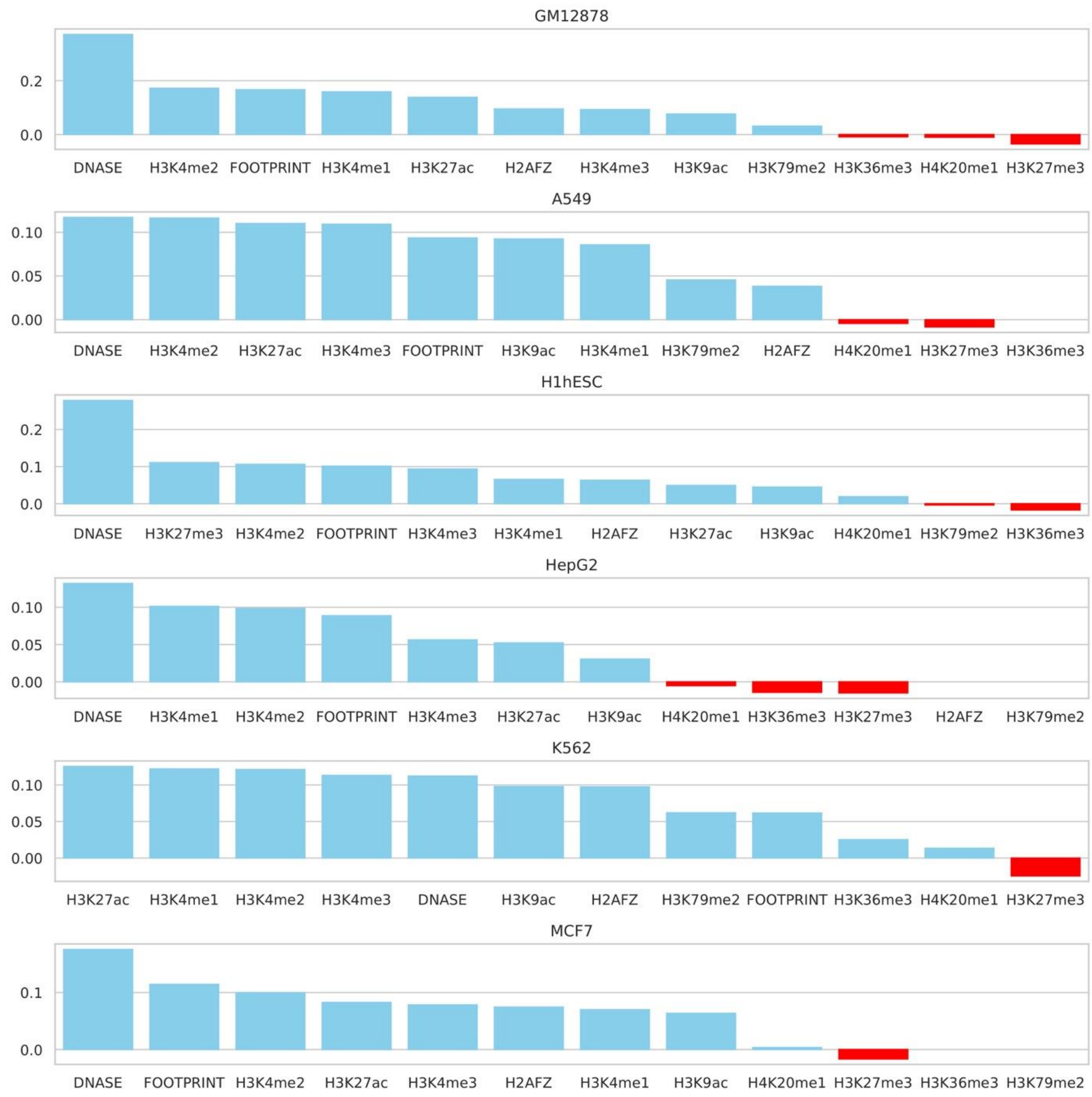

Supplementary Figure 3. Pearson correlation of labels and tissue-specific features in 6 ASB datasets. Blue bars represent positive correlations while red bars represent negative correlations.

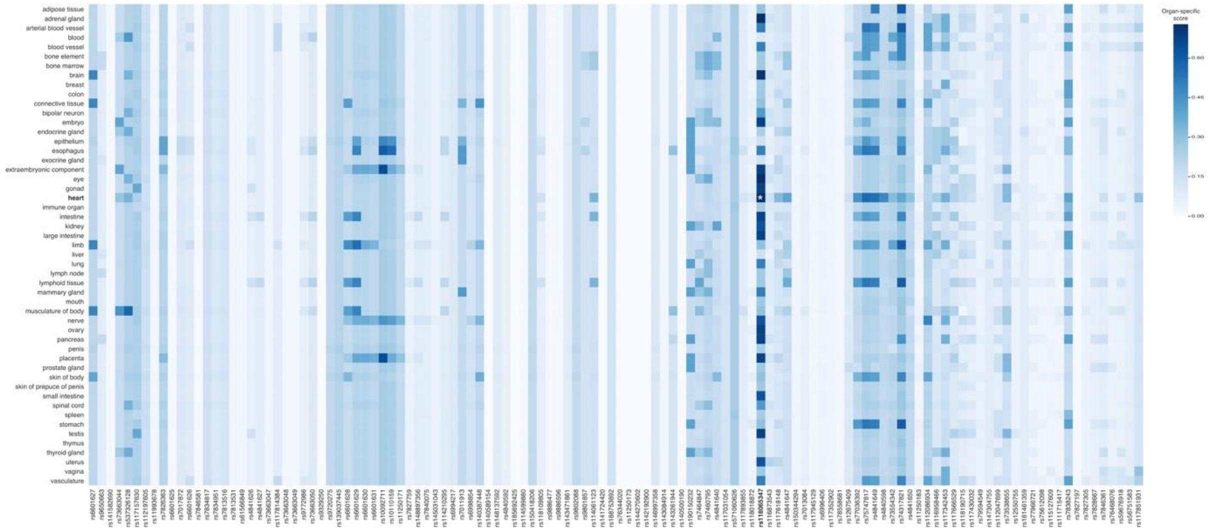

Supplemental Figure S4. Organ-specific scores of variants in the *GATA4* locus. rs118065347 has the top organ-specific score in heart compared to other candidate SNPs found through genome-wide association scan on bicuspid aortic valve cases. rs118065347 was shown to have hear-specific regulatory functionality from a previous study Yang et al. 2017.

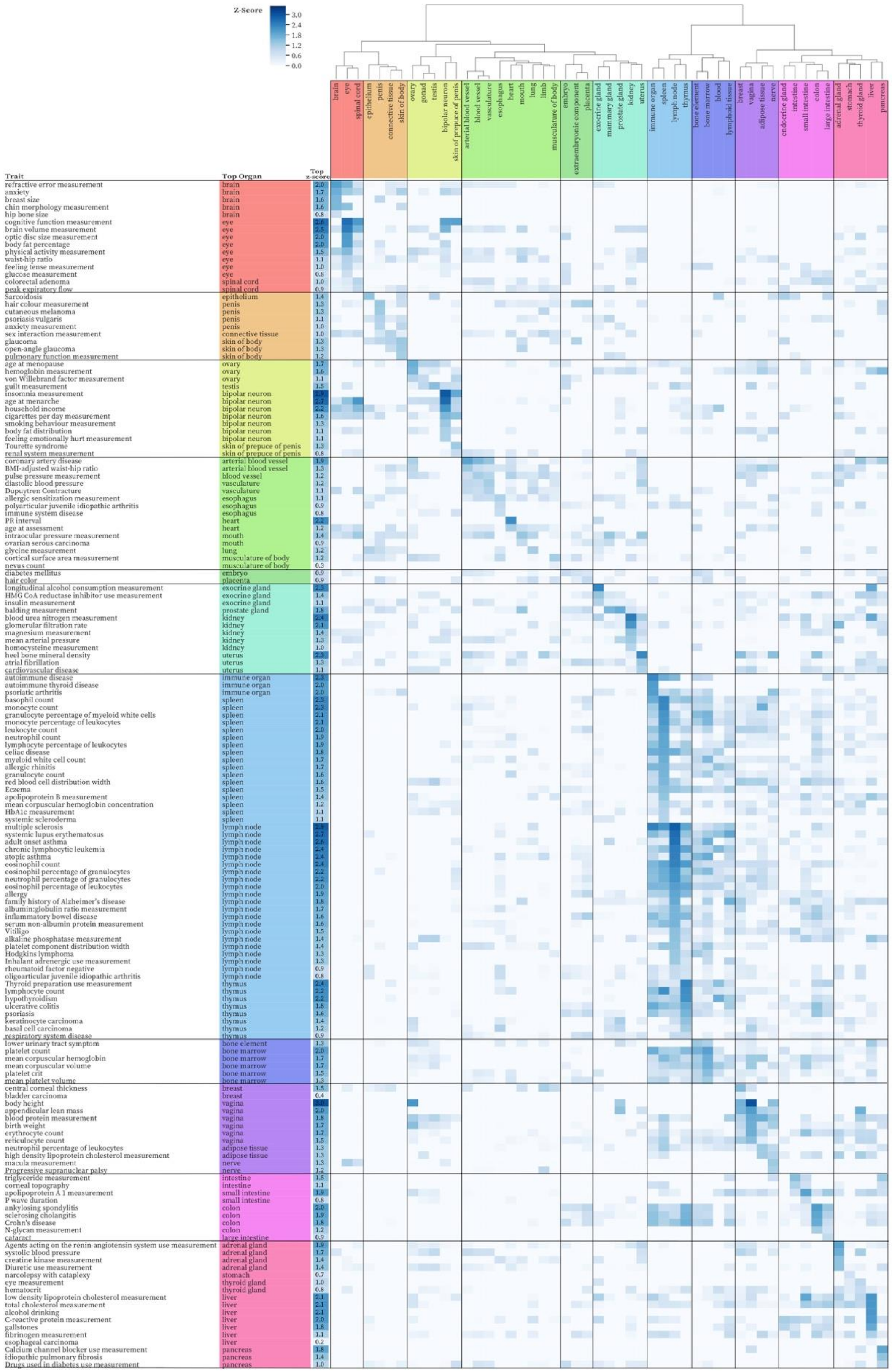
Z-Score
3.0
2.4
1.8
1.2
0.6
0.0
brain
eye
spinal cord
epithelium
penis
connective tissue
skin of body
ovary
gonad
testis
bipolar neuron
skin of prepuce of penis
arterial blood vessel
blood vessel
vasculature
esophagus
heart
mouth
lung
limb
musculature of body
embryo
extraembryonic component
placenta
exocrine gland
mammary gland
prostate gland
kidney
uterus
immune organ
spleen
lymph node
thymus
bone element
bone marrow
blood
lymphoid tissue
breast
vagina
adipose tissue
nerve
endocrine gland
intestine
small intestine
colon
large intestine
adrenal gland
stomach
thyroid gland
liver
pancreas
Trait
Top Organ
Top z-score
refractive error measurement brain 2.0
anxiety brain 1.7
breast size brain 1.6
chin morphology measurement brain 1.6
hip bone size brain 0.8
cognitive function measurement eye 2.6
brain volume measurement eye 2.5
optic disc size measurement eye 2.0
body fat percentage eye 2.0
physical activity measurement eye 1.5
waist-hip ratio eye 1.1
feeling tense measurement eye 1.0
glucose measurement eye 0.8
colorectal adenoma spinal cord 1.0
peak expiratory flow spinal cord 0.9
Sarcoidosis epithelium 1.4
hair colour measurement penis 1.3
cutaneous melanoma penis 1.3
psoriasis vulgaris penis 1.1
anxiety measurement penis 1.0
sex interaction measurement connective tissue 1.0
glaucoma skin of body 1.3
open-angle glaucoma skin of body 1.3
pulmonary function measurement skin of body 1.2
age at menopause ovary 1.7
hemoglobin measurement ovary 1.6
von Willebrand factor measurement ovary 1.1
guilt measurement testis 1.5
insomnia measurement bipolar neuron 2.9
age at menarche bipolar neuron 2.7
household income bipolar neuron 2.2
cigarettes per day measurement bipolar neuron 1.6
smoking behaviour measurement bipolar neuron 1.3
body fat distribution bipolar neuron 1.1
feeling emotionally hurt measurement bipolar neuron 1.1
Tourette syndrome skin of prepuce of penis 1.3
renal system measurement skin of prepuce of penis 0.8
coronary artery disease arterial blood vessel 1.9
BMI-adjusted waist-hip ratio arterial blood vessel 1.3
pulse pressure measurement blood vessel 1.2
diastolic blood pressure vasculature 1.2
Dupuytren Contracture vasculature 1.1
allergic sensitization measurement esophagus 1.1
polyarticular juvenile idiopathic arthritis esophagus 0.9
immune system disease esophagus 0.8
PR interval heart 2.2
age at assessment heart 1.2
intraocular pressure measurement mouth 1.4
ovarian serous carcinoma mouth 0.9
glycine measurement lung 1.2
cortical surface area measurement musculature of body 1.2
nevus count musculature of body 0.3
diabetes mellitus embryo 0.9
hair color placenta 0.9
longitudinal alcohol consumption measurement exocrine gland 2.3
HMG CoA reductase inhibitor use measurement exocrine gland 1.4
insulin measurement exocrine gland 1.1
balding measurement prostate gland 1.8
blood urea nitrogen measurement kidney 2.4
glomerular filtration rate kidney 2.1
magnesium measurement kidney 1.4
mean arterial pressure kidney 1.3
homocysteine measurement kidney 1.0
heel bone mineral density uterus 2.3
atrial fibrillation uterus 1.3
cardiovascular disease uterus 1.1
autoimmune disease immune organ 2.3
autoimmune thyroid disease immune organ 2.0
psoriatic arthritis immune organ 2.0
basophil count spleen 2.3
monocyte count spleen 2.3
granulocyte percentage of myeloid white cells spleen 2.1
monocyte percentage of leukocytes spleen 2.1
leukocyte count spleen 2.0
neutrophil count spleen 1.9
lymphocyte percentage of leukocytes spleen 1.9
celiac disease spleen 1.8
myeloid white cell count spleen 1.7
allergic rhinitis spleen 1.7
granulocyte count spleen 1.6
red blood cell distribution width spleen 1.6
Eczema spleen 1.5
apolipoprotein B measurement spleen 1.4
mean corpuscular hemoglobin concentration spleen 1.2
HbA1c measurement spleen 1.1
systemic scleroderma spleen 1.1
multiple sclerosis lymph node 2.9
systemic lupus erythematosus lymph node 2.7
adult onset asthma lymph node 2.6
chronic lymphocytic leukemia lymph node 2.4
atopic asthma lymph node 2.4
eosinophil count lymph node 2.4
eosinophil percentage of granulocytes lymph node 2.2
neutrophil percentage of granulocytes lymph node 2.2
eosinophil percentage of leukocytes lymph node 2.0
allergy lymph node 1.9
family history of Alzheimer's disease lymph node 1.8
albumin:globulin ratio measurement lymph node 1.7
inflammatory bowel disease lymph node 1.6
serum non-albumin protein measurement lymph node 1.6
Vitiligo lymph node 1.5
alkaline phosphatase measurement lymph node 1.4
platelet component distribution width lymph node 1.4
Hodgkins lymphoma lymph node 1.3
Inhalant adrenergic use measurement lymph node 1.3
rheumatoid factor negative lymph node 0.9
oligoarticular juvenile idiopathic arthritis lymph node 0.8
Thyroid preparation use measurement thymus 2.4
lymphocyte count thymus 2.2
hypothyroidism thymus 2.2
ulcerative colitis thymus 1.8
psoriasis thymus 1.6
keratinocyte carcinoma thymus 1.4
basal cell carcinoma thymus 1.2
respiratory system disease thymus 0.9
lower urinary tract symptom bone element 1.3
platelet count bone marrow 2.0
mean corpuscular hemoglobin bone marrow 1.7
mean corpuscular volume bone marrow 1.7
platelet crit bone marrow 1.5
mean platelet volume bone marrow 1.3
central corneal thickness breast 1.5
bladder carcinoma breast 0.4
body height vagina 3.0
appendicular lean mass vagina 2.0
blood protein measurement vagina 1.8
birth weight vagina 1.7
erythrocyte count vagina 1.7
reticulocyte count vagina 1.5
neutrophil percentage of leukocytes adipose tissue 1.3
high density lipoprotein cholesterol measurement adipose tissue 1.3
macula measurement nerve 1.3
Progressive supranuclear palsy nerve 1.2
triglyceride measurement intestine 1.5
corneal topography intestine 1.1
apolipoprotein A 1 measurement small intestine 1.9
P wave duration small intestine 0.8
ankylosing spondylitis colon 2.0
sclerosing cholangitis colon 1.9
Crohn's disease colon 1.8
N-glycan measurement colon 1.2
cataract large intestine 0.9
Agents acting on the renin-angiotensin system use measurement adrenal gland 1.9
systolic blood pressure adrenal gland 1.7
creatine kinase measurement adrenal gland 1.4
Diuretic use measurement adrenal gland 1.4
narcolepsy with cataplexy stomach 0.7
eye measurement thyroid gland 1.0
hematocrit thyroid gland 0.8
low density lipoprotein cholesterol measurement liver 2.1
total cholesterol measurement liver 2.1
alcohol drinking liver 2.1
C-reactive protein measurement liver 2.0
gallstones liver 1.8
fibrinogen measurement liver 1.1
esophageal carcinoma liver 0.2
Calcium channel blocker use measurement pancreas 1.8
idiopathic pulmonary fibrosis pancreas 1.4
Drugs used in diabetes use measurement pancreas 1.0

Supplemental Figure S5. Enrichment of regulatory variants with high organ-specific scores over variants associated with diverse traits. Only organ-trait pairs with z-scores higher than 0 and passing multiple test correction (FDR threshold of 5%) are shown.

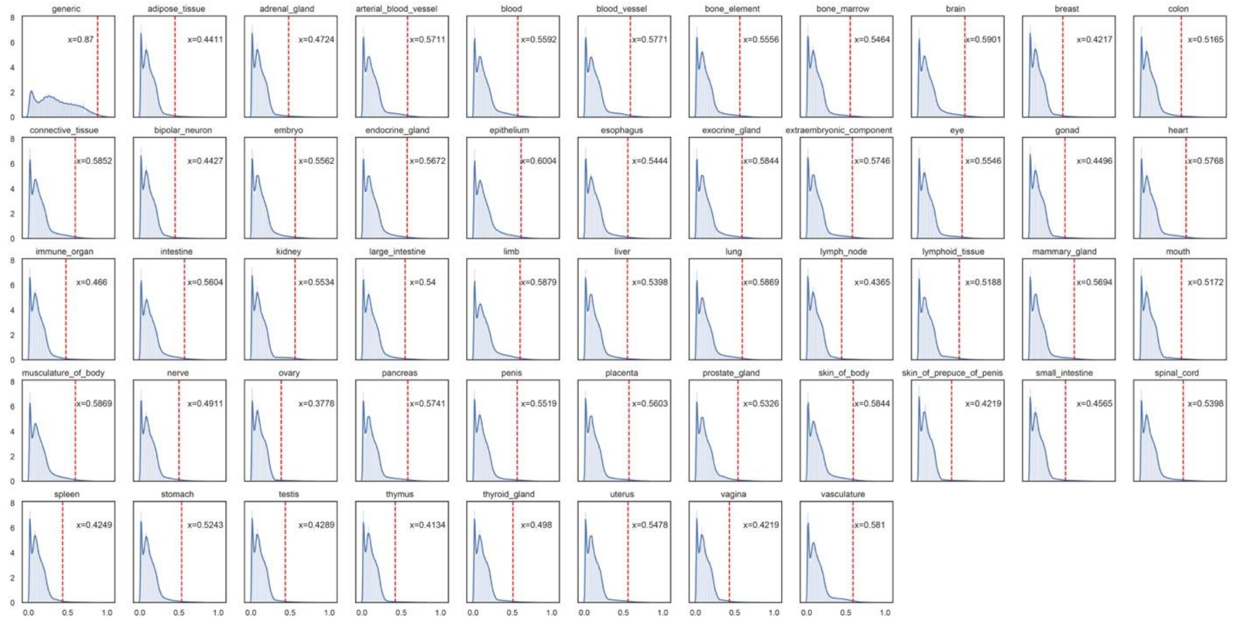

Supplemental Figure S6. Distribution of TURF generic and organ-specific scores on 3,102,261 GM12878 SNVs. The red vertical dashed line represents the 99th percentile score in each subplot.
